# Bone Marrow Mesenchymal Stem-Cell Transplantation Promotes Functional Improvement Associated with CNTF-STAT3 Activation after Hemi-Sectioned Spinal Cord Injury in Tree Shrews

**DOI:** 10.3389/fncel.2017.00172

**Published:** 2017-06-28

**Authors:** Liu-Lin Xiong, Fei Liu, Bing-Tuan Lu, Wen-Ling Zhao, Xiu-Juan Dong, Jia Liu, Rong-Ping Zhang, Piao Zhang, Ting-Hua Wang

**Affiliations:** ^1^Institute of Neurological Disease and Department of Anesthesiology, Translational Neuroscience Center, West China Hospital, Sichuan UniversityChengdu, China; ^2^Institute of Neuroscience, Animal Zoology Department, Kunming Medical UniversityKunming, China; ^3^Key Laboratory of National Physical Fitness and Altitude Training Adaptation in Yunnan Province, Institute of Physical Education, Yunnan Normal UniversityKunming, China; ^4^Biomedical Engineering Research Center, Kunming Medical UniversityKunming, China

**Keywords:** tree shrews, bone marrow mesenchymal stem cells, spinal cord injury, cell transplantation, STAT3, CNTF, cell apoptosis

## Abstract

Hemi-sectioned spinal cord injury (hSCI) can lead to spastic paralysis on the injured side, as well as flaccid paralysis on the contralateral side, which can negatively affect a patient’s daily life. Stem-cell therapy may offer an effective treatment option for individuals with hSCI. To examine the role of bone marrow mesenchymal stem cells (BMSCs) transplantation on hSCI and explore related mechanisms in the tree shrews, here, we created a model of hSCI by inducing injury at the tenth thoracic vertebra (T10). Hoechst 33342-labeled BMSCs derived from adult tree shrews were isolated, cultured, and implanted into the spinal cord around the injury site at 9 days after injury. The isolated BMSCs were able to survive, proliferate and release a variety of neurotrophic factors (NTFs) both *in vitro* and *in vivo*. At 28 days after injury, compared with the sham group, the hSCI group displayed scar formation and dramatic elevations in the mean interleukin 1 beta (IL-1β) density and cell apoptosis level, whereas the expression of signal transducer and activator of transcription 3 (*STAT3*) and ciliary neurotrophic factor (*CNTF*) mRNA was reduced. Following BMSC transplantation, motoneurons extent of shrinkage were reduced and the animals’ Basso, Beattie, and Bresnahan (BBB) locomotion scale scores were significantly higher at 21 and 28 days after injury when compared with the injured group. Moreover, the hSCI-induced elevations in scar formation, IL-1β, and cell apoptosis were reduced by BMSC transplantation to levels that were close to those of the sham group. Corresponding elevations in the expression of *STAT3* and *CNTF* mRNA were observed in the hSCI + BMSCs group, and the levels were not significantly different from those observed in the sham group. Together, our results support that grafted BMSCs can significantly improve locomotor function in tree shrews subjected to hSCI and that this improvement is associated with the upregulation of *CNTF* and *STAT3* signaling.

## Introduction

Spinal cord injury (SCI) is often associated with a low quality of life and high mortality (Zompa et al., 1997; Han et al., 2015). Currently, treating SCI is a challenge, as existing therapies are expensive and provide unsatisfactory functional improvement. Traditionally, it was believed that the nerves in the central nervous system (CNS) of mature mammals could not regenerate after injury owing to the poor regenerative capability of neurons and inhibition from the glial microenvironment. However, Aguayo et al. (1981) discovered that injured axons in the CNS could regenerate in a suitable environment, thus fostering the development of new approaches for revealing the mechanisms underlying this regeneration and repair, with the aim of establishing optimal therapeutic strategies for individuals with SCI.

Cell transplantation, as a method of biotherapy, is an effective treatment method for neurological diseases (Chopp et al., 2000; Hofstetter et al., 2002; Wu et al., 2003). Bone marrow mesenchymal stem cells (BMSCs), multipotent stromal cells that are derived from bone marrow (Méndez-Ferrer et al., 2015), support hematopoiesis and generate cells in the mesodermal layers (Garfias et al., 2012). Recently, the administration of BMSCs has been shown to downregulate T and B lymphocytes, natural killer cells, and antigen-presenting cells by cell-to-cell interactions and soluble factor production (Kassis et al., 2011). Besides their immunomodulatory effects, BMSCs also have the potential for self-renewal and multipotency and can secrete neurotrophic factors (NTFs; Méndez-Ferrer et al., 2015). These immunomodulatory and neuroprotective features imply that BMSCs may serve as potential candidates for future therapeutic modalities in SCI.

Numerous previous studies have shown that BMSC transplantation dose-dependently promotes functional improvement and even alleviates the symptoms of neuropathic pain during or after the spinal shock period in rat, dog, rabbit, monkey and even human models of SCI (Pal et al., 2010; Amr et al., 2014; Mendonça et al., 2014; Torres-Espín et al., 2014; Xia et al., 2014; Han et al., 2015; Satti et al., 2016; Yousefifard et al., 2016). The mechanisms underlying the ability of BMSCs to promote functional remodeling after SCI are likely related to the anti-proliferative and anti-apoptotic proprieties of these cells, which exert anti-inflammatory and immunosuppressive effects at the injury site; additionally, BMSCs induce the repair of nerve cells, promote axonal regeneration, and restore nerve trophism by secreting NTFs (Ohta et al., 2004; Neuhuber et al., 2005; Abrams et al., 2009; Sakata et al., 2011; Tran et al., 2011; Uccelli et al., 2011; Xia et al., 2014; Abbaszadeh et al., 2015; Han et al., 2015). These effects translate into functional improvement, as a study using a dog model of SCI demonstrated that both autologous and allogenic BMSC transplantation improved neurological function following SCI and noted that these improvements were associated with reduced interleukin-6 (IL-6) and cyclooxygenase-2 levels (Jung et al., 2009; Ryu et al., 2012). These findings support that BMSC transplantation may be an effective treatment for SCI. However, most prior studies have focused on BMSCs derived from rats, while few investigations have examined the role of BMSC in primate models of SCI. Therefore, new animal models that more closely resemble humans are needed.

The tree shrews (*Tupaia belangeri*) are small mammals that are mainly distributed in the Torrid Zone and subtropical zone of Southeast Asia (Peng et al., 1991). Because of a close relationship to primates in terms of the biological characteristics, biochemistry, metabolism, physiology and genome (Pawlik et al., 1999; Cao et al., 2003; Janecka et al., 2007; Fan et al., 2013, 2014), the tree shrew has got wide attention. Moreover, the tree shrews has been increasingly used as a viable model animal, and are proposed to be an alternative experimental animal to rodents and primates in biomedical research, as they have several characteristics including low cost of maintenance, small adult body size, high brain-to-body mass ratio, a short reproductive cycle, and can be more easily obtained than monkeys (Cao et al., 2003; Fuchs and Corbach-Söhle, 2010; Fan et al., 2013, 2014; Xu et al., 2013). In addition to Alzheimer’s disease (Yamashita et al., 2010; Lin et al., 2016), the tree shrew has also been successfully used as an animal model of liver disease (Zhao et al., 2002), depression (Wang et al., 2011), myopia (Norton et al., 2006), bacterial infection (Li et al., 2012) and learning behaviors (Bartolomucci et al., 2002). Hence, the tree shrew has the potential to serve as a viable model for cell transplantation in translational studies examining SCI.

The aim of the present study was to examine the effects of BMSC transplantation in a tree shrew model of SCI. To do this, we cultured BMSCs that were isolated from the healthy tree shrews *in vitro* and evaluated their morphology, proliferation and NTF expression levels, including brain-derived neurotrophic factor (BDNF), transforming growth factor beta 1 (TGF-β1), and ciliary neurotrophic factor (CNTF). Hemi-transected spinal cord injury (hSCI) was then induced in tree shrews. Subsequently, the Hoechst 33342-labeled BMSCs were transplanted into the injured spinal cord to determine their *in vivo* effects on locomotor function in the hind limbs, glial scar formation, the inflammatory response, cell apoptosis and motoneurons. Lastly, the underlying mechanisms involved in these effects were determined.

## Materials and Methods

### Animals and Groups

Thirty-three healthy adult female tree shrews weighing 100 ± 10 g were provided by the Experimental Animal Center of Kunming Medical University (Kunming, China). Experiment protocol was legally approved by the Animal Care and Welfare Committee of Kunming Medical University. All experiments in the animals also conformed to the guidelines for laboratory animal care and safety as issued by the United States National Institutes of Health. Animals were housed in individual cages in a temperature- (20 ± 5°C) and humidity (40%–60%)-controlled room with a 12-h light/dark cycle, they were free access to pellet chow and water. As shown in Table 1, all tree shrews were randomly divided into three groups.

**Table 1:** Animal grouping and sample used in each group.

Group	qRT-PCR (28 dpi)	IF/IHC/TUNEL (28 dpi)
	Behavior (1, 3, 5, 7, 9, 11, 13 dpi)	Behavior (15, 17, 19, 21, 23, 25, 28 dpi)
	(*n*)	(*n*)
hSCI (*n* = 10)	5	5
hSCI + BMSCs (*n* = 10)	5	5
Sham (*n* = 10)	5	5

*hSCI, hemi-sectioned spinal cord injury with culture medium; qRT-PCR, quantitative reverse transcription-polymerase chain reaction; IF, immunofluorescence; dpi, days post injury; TUNEL, Terminal-deoxynucleoitidyl Transferase Mediated Nick End Labeling; IHC, immunohistochemistry*.

### *In Vitro* Study

#### BMSCs Culture and Purification

BMSCs were harvested from the femurs and tibias of three tree shrews as described in previous reports (Hofstetter et al., 2002; Fu et al., 2015). Briefly, the tree shrews were anesthetized intraperitoneally by 2% pentobarbital sodium (30 mg/kg), the femurs and tibias were then dissected in a sterile environment and rinsed with D-Hanks. Epiphyses of the femurs were removed, and the marrow was then extruded using a syringe filled with DMEM/F12 containing 10% fetal bovine serum (FBS, Gibco), and repeatedly beated into a single cell suspension with 5 ml DMEM/F12 containing 10% FBS and penicillin/streptomycin. After centrifuging (1000 r/min, 5 min) and re-suspending, the cells were plated in 75 cm^2^ culture flasks at a density of 1 × 10^6^/ml in an incubator (37°C, 95% humidity, 5% CO_2_). Twelve hours after incubation at 37°C and 5% CO_2_, supernatant containing nonadherent cells were removed and fresh medium was added. Medium was changed twice a week, when cells were about (4–5) × 10^6^ cells/cm^2^, they were passaged 2–4 times and the suspension cells were discarded. The adherent cells were continually cultivated. Therefore, we got the pure adherent BMSC for further study.

#### Morphology and Proliferation of BMSCs

The morphology and proliferation of BMSC (passage four) were observed at day 3, day 5, day 9 and day 12 under an inverted phase contrast microscope (Leica Microsystems Wetzlar GmbH). Cell number (from five fields in each well and five wells of each day) was then calculated for BMSC proliferation evaluation using Image-Pro plus 6.0 software (Media Cybernetics, Silver Spring, MD, USA).

#### Identification, Differentiation of BMSCs and Expression of NTFs *In Vitro*

In order to identify the cultured BMSC and detect the expression of NTFs, corresponding primary and secondary antibodies (Table 2) for immunofluorescent staining were used following a previously reported protocol (Liu et al., 2014). Negative control was incubated with phosphate buffer saline (PBS) to replace the primary antibody. Thereafter, DAPI was used to label the BMSC nucleus; the cells were photographed under the fluorescent microscope (Leica, Germany). Then, the positive rate (mean number of the positive cells/the number of DAPI) was calculated by three investigators blinded to the experimental information using Image-Pro plus 6.0 software (Media Cybernetics, Silver Spring, MD, USA).

**Table 2:** Primary and secondary antibodies used in immunofluorescence.

First antibody	Company	Nature	Concentration	Secondary antibody	Company	Nature	Concentration
CD44	Bioss	rabbit	1:100	Cy3	Jackson	goat anti-rabbit	1:200
CD29	Bioss	rabbit	1:100	Cy3	Jackson	goat anti-rabbit	1:200
CD45	Bioss	rabbit	1:100	Cy3	Jackson	goat anti-rabbit	1:200
GFAP	Zhongshan	rabbit	1:50	Cy3	Jackson	goat anti-rabbit	1:200
Tuj1	Zhongshan	rabbit	1:100	Cy3	Jackson	goat anti-rabbit	1:200
BDNF	Boster	rabbit	1:50	Cy3	Jackson	goat anti-rabbit	1:200
TGFβ1	Abcam	rabbit	1:100	Cy3	Jackson	goat anti-rabbit	1:200
CNTF	Zhongshan	rabbit	1:50	Cy3	Jackson	goat anti-rabbit	1:200

*GFAP, glial fibrillary acidic protein; Tuj1, Neuronal Class III β-Tubulin; CD44, CD 29, CD45 are the specific surface markers of BMSC, to identify the BMSC; Tuj1 is the specific marker for neurons; BDNF, brain-derived neurotrophic factor; TGF-β1, transforming growth factor beta 1; CNTF, ciliary neurotrophic factor*.

#### Western Blot

Western blot was used to confirm the true expression of neurotrophins, and detailed protocols were described in a previous report (Liu et al., 2014). Briefly, protein was extracted from BMSC samples, after the protein concentration was certained by BCA protein assay kit (Beyotime Institute), 60 μg samples were separated onto 4%–12% gradient gels and transferred onto a polyvinylidene difluoride (PVDF) membrane. The membranes were then blocked with 5% nonfat milk in TBST for 2 h at room temperature and incubated overnight at 4°C with related primary (Table 3) antibodies, which was followed by incubation with corresponding secondary antibodies (anti-mouse/anti-rabbit, Abcam, 1:5000) for 2 h at room temperature. β-actin was used as a loading control. Afterwards, the immunoblot was revealed with an ECL Western Blot Detection Kit (Amersham Pharmacia Biotech, Buckinghamshire, England). Densitometry analysis was performed using ImageJ software.

**Table 3:** Primary antibodies in western blot.

Primary antibody	Dilution	Nature	Company
CNTF	1:500		ZSGB-BIO,
TGFβ1	1:1000	Rat	Beijing,
BDNF	1:1000		China
β-actin	1:1000	Mouse	Abcam

*BDNF, brain-derived neurotrophic factor; TGF-β1, transforming growth factor beta 1; CNTF, ciliary neurotrophic factor*.

### *In Vivo* Study

#### hSCI Model

All tree shrews were anesthetized intraperitoneally by 2% pentobarbital sodium (30 mg/kg) and placed in the prone position. A midline skin incision was made at the thoracic area (T8–T12), then paravertebral muscles and supraspinal ligaments were separated to expose the T10 spinal segment. Subsequently, the spinal cord at T10 was hemisected by microscissors to establish the tree shrew hSCI. Sham group underwent all the procedures except the T10 hemisection. Then, the surgical wounds were closed with a 3–0 silk suture and the tree shrews were injected intraperitoneally with 5% cefotaxime sodium salt (diluted 10× with normal saline) (0.5 μl per tree shrew) once a day for 7 days. Their bladders were manually massaged three times a day until recovery of micturition reflex.

#### BMSC Labeling and Transplantation

According to the cell morphology and proliferation, the BMSCs at passage four were collected when they reached 90% of confluence at day 12 in culture and selected for transplantation. In addition, for tracing cells, these BMSCs were stained with Hoechst 33342 (Beyotime Biotechnology, China) for 5 min 2 h before transplantation. Cell morphology was directly observed under a fluorescent microscope (Leica, Germany), then the cells were counted and concentrated with a final concentration of 2.0 × 10^4^/μl.

At 9 days after hSCI (McDonald et al., 1999; Alexanian et al., 2011), each tree shrew was anesthetized as described above and placed in a stereotaxic frame, then the T10 spinal segment was exposed as previously described. Cell suspension was implanted into the spinal cord by the insertion of a pored glass pipette attached to a micro-injector carrying a capillary glass microelectrode (Thermo Scientific, Rockford, IL, USA). Six sites with 2 mm depth were selected at the following coordinates: two sites 5 mm rostral to the injured site, two sites 5 mm caudal to the injured site, one site 0.5 mm left and one site 0.5 mm right to the injured site. Each site was injected with approximately 2 μl cell suspension. Infusion was performed at a rate of 600 nl/min. After injection, the glass pipette was left for an additional 5 min before being slowly retracted. The control group underwent the same procedure using equivalent culture medium. In addition, cyclosporin-A was intraperitoneally (10 mg/kg per day) used from the third day before cell transplantation, and kept till the animals were sacrificed to suppress the immunoreaction. This could provide an environment for BMSCs survival in the host spinal cord. In order to exclude possible effects of immunosuppression on experimental outcome such as signal transducer and activator of transcription 3 (STAT3) mRNA levels, all the tree shrews in the sham, hSCI and hSCI + BMSCs groups received the same cyclosporin-A treatment.

#### Behavioral Evaluation

At day 1–28 after hSCI, hindlimb motor function of the tree shrews in three groups was assessed with the open-field Basso, Beattie, and Bresnehan (BBB) scoring system, which was graded from 0 point (absence of any hind limb movement) to 21 points (normal mobility). In brief, subjects were acclimated to the open enclosure (99 cm in diameter, 23 cm deep) for 3 days prior to detection, 5 min per day. Then, each subject was observed for 4 min. The final score was the average of these three individual researchers (Basso et al., 1995).

#### Sample Harvest and Observation of BMSCs in Spinal Cord Samples

At 28 days after injury, all tree shrews were sacrificed and spinal cords caudal to the injured site (10-mm-long containing injury and graft) were removed for later use. Spinal cords for immunohistochemical and terminal-deoxynucleoitidyl transferase mediated nick end labeling (TUNEL) observation were dehydrated by 30% sucrose overnight; then, they were sectioned at 10-μm thickness in a freezing microtome (Leica CM1900, Germany). For survival detection, the spinal cord tissue near the injured site was (1 cm below the lesion) harvested, and sectioned into10-μm thickness for later observation under the fluorescent microscope. In addition, the spinal cords caudal to the injured site from the other animals (10-mm-long containing injury and graft) for quantitative real-time PCR (qRT-PCR) were harvested and stored in 1.5 ml EP tube without RNase at −80°C until further required. The sample arrangement was showed in Table 1.

#### Immunofluorescence

For observing the inflammatory reaction, immunofluorescent staining of interleukin-l β (IL-1β) was performed. In brief, the primary antibody of IL-1β (1:200, Rabbit, Santa), as well as the secondary antibody of Alexa 594 (goat anti-rabbit, 1:200, Zhongshan golden bridge) were applied progressively as previously described (Liu et al., 2014, 2015). Negative control was performed by omitting the primary antibody. Slides were then photographed using a fluorescent microscope (Leica, Germany). The mean density of IL-1β was presented as IOD (integrated optical density) over the total (mm^2^) area and quantified using Image-Pro plus 6.0 software (Media Cybernetics, Silver Spring, MD, USA; Sun et al., 2013).

#### Terminal-deoxynucleoitidyl Transferase Mediated Nick End Labeling (TUNEL) Assay

TUNEL assay was performed as described in one previous report (He et al., 2016). In brief, a TUNEL reaction mixture of enzyme solution and labeling solution (*in situ* Cell Death Detection Kit, TMR red; Cat.NO.12156792910) was added at a ratio of 1:9 (v/v). Then the slices were incubated at 4°C overnight in the dark and stained with DAPI for 5 min at room temperature followed by three washes with PBS. Subsequently, pictures were obtained under the fluorescent microscopy (Leica, CM1860, Germany). Finally, cell apoptosis was quantified by calculating the percentage of TUNEL/DAPI using Image-Pro plus 6.0 software.

#### Enzyme Histochemical Staining

Enzyme histochemistry was used to observe the glial scar formation (glial fibrillary acidic protein (GFAP) immunohistochemical staining) and motoneurons from lamina IX (Neuronal Nuclei (NeuN) immunohistochemical staining) in the spinal cord tissues. Following three washes in 0.01 mol/L PBS, the slices were incubated with 3% hydrogen peroxide at 37°C for 15 min to block the action of any endogenous peroxidase. Then the sections were incubated with 5% goat serum at 37°C for 30 min. Continually, the tissue sections were incubated overnight at 4°C with GFAP (1:100, rabbit, Bioss) and NeuN (1:100, rabbit, Bioss) primary antibodies respectively. PBS was served as the negative control. Afterwards, sections were separately incubated with PV-9000 reagent 1 (Zhongshan golden bridge) and PV-9000 reagent 2 (Zhongshan golden bridge) for 30 min at 37°C. Subsequently, DAB was incubated as chromogenic agent for 3–7 min in the dark and the sections were counter-stained with hematoxylin. Finally, the positive staining was visualized under an inverted phase contrast microscope imaging system (five sections/animal), the cell area and mean density of GFAP (IOD/area) were analyzed using Image-Pro Plus 6.0 software (Media Cybernetics, Silver Spring, MD, USA) as above described.

#### QRT-PCR

At 28 days after injury, spinal cord caudal to the injured site (10-mm-long containing injury and graft) from BMSC, hSCI and sham groups were collected and homogenized to determine the level of mitogen-activated protein kinase kinase *(Merk), phosphatidylinositol 3-kinase (PI3K), STAT3* and *CNTF* mRNA. Briefly, total RNA was isolated with Trizol reagent (Takara Bio Inc., Otsu, Japan) and was reverse transcribed to cDNA with the Revert Aid™ First Strand cDNA Synthesis kit (Takara Biotechnology, Co., Ltd., Dalian, China). Subsequently, qRT-PCR of cDNA was performed using the specific primers, which were synthesized by TaKaRa Company (Japan), the sequences were described in Table 4. β-actin was used as the internal control. PCR cycling conditions were as follows: initial denaturation at 95°C for 2 min, denaturation at 95°C for 15 s and amplification at 53°C for 20 s, followed by extension 60°C for 30 s for a total of 40 cycles. The threshold cycle (Ct) of each sample was recorded, and data were analyzed by normalization to β-actin values using the 2^−ΔΔCt^ method (Livak and Schmittgen, 2001; Liu et al., 2014).

**Table 4:** Information of primer sequences.

Gene	Upstream	Downstream	Annealing temperature(°C)
Merk	5′-GGCTTCTATGGTGCGTTCTA-3′	5′-AGGATGTTGGAGGGCTTGAC-3′	51
PI3K	5′-GCTTGCTGTCTCCTCTAAACCCTG-3′	5′-CGCACCACCTCAATAAGTCCC-3′	51
STAT3	5′-TTTTGTCAGCGATGGAGTA-3′	5′-TTGTTGACGGGTCTGAAGT-3′	51
CNTF	5′-CCAGGCTCTTAGAAGACCAG-3′	5′-AGACCACCATCTCCAACATTA-3′	53
β-actin	5′-GAAGATCAAGATCATTGCTCCT-3′	5′-TACTCCTGCTTGCTGATCCA-3′	52

*CNTF, ciliary neurotrophic factor; Merk, mitogen-activated protein kinase kinase; PI3K, phosphatidylinositol 3-kinase; STAT3, signal transducer and activator of transcription 3*.

#### Statistical Analysis

Experimental results are expressed as the mean ± standard deviation (SD) and analyzed by one-way ANOVA using SPSS software (version 19.0, SPSS Inc., Chicago, IL, USA). The data had significant differences when *p* < 0.05.

## Results

### Proliferation of BMSCs *In Vitro*

**Figure 1:**
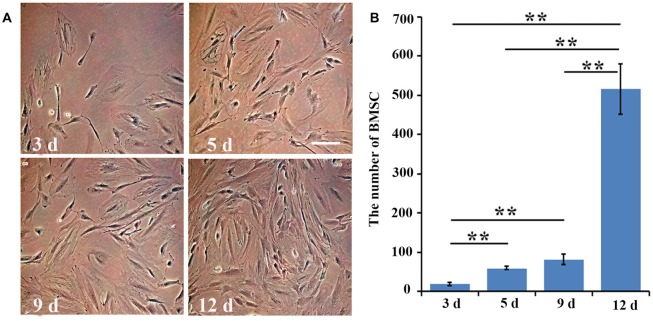
The number of bone marrow mesenchymal stem cells (BMSCs) at different time points. **(A)** The cultured BMSCs at day 3, day 5, day 9 and day 12 at passage four were observed under the inverted phase contrast microscope. **(B)** Quantitative analysis for the proliferation of cultured BMSCs at day 3, day 5, day 9 and day 12. Data are presented as the mean ± standard deviation (SD) (*n* = 5). **p < 0.01 for the comparison of the two time points below the straight line. Bar = 100 μm.

At day 3, the morphology of the cultured BMSCs at passage four was normal. As for their proliferation, the number of cells was significantly (three fold) higher at day 5 than it was at day 3 (*p* = 0.006). At day 9, the number of BMSCs was slightly higher than that at day 5 (*p* = 0.08), and was about four times higher than that at day 3 (*p* = 0.003). The cultured BMSCs completely covered the bottom of the flasks and exhibited the strongest proliferation ability at day 12, with the number of cells being approximately five or six times higher than that at day 9 (*p* = 0.002; Figures 1A,B). Hence, the BMSCs at passage four and at day 12 in culture were selected for the further transplant study.

### Identification of BMSCs *In Vitro*

**Figure 2:**
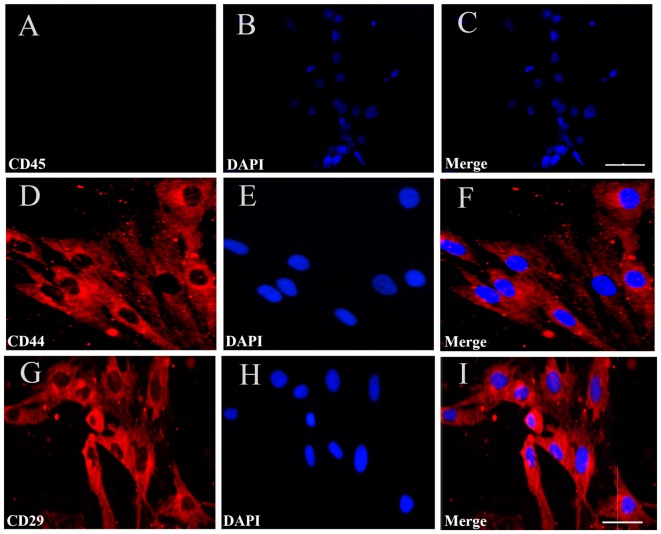
Immunofluorescent staining for BMSCs identification. **(A)** Immunofluorescent staining of CD45 in the cultured BMSCs, but no cells expressed CD45. **(D,G)** There were CD44 and CD29 red positive immunofluorescence (IF). **(B,E,H)** Cell nucleus was stained by DAPI with blue fluorescence. **(C,F,I)** The merged images showed the mean positive rate of CD44, CD29and CD45 were about 98.8%, 99.3% and 0%. Bar = 50 μm in **(A–C)**, 25 μm in **(D–I)**. The mean positive rate represented as mean number of the positive cells/the number of DAPI was analyzed using Image-Pro plus 6.0 software. CD44, CD 29, CD45 are the specific surface markers to identify the BMSC.

To identify the cultured cells, at passage four, we performed immunofluorescent staining for CD44, CD45 and CD29, which are specific surface markers of BMSCs. The results showed that 98.8% and 99.3% of the cells were positive for CD44 and CD29, respectively, while the cells were negative for CD45, a distinctive molecule found in hemopoietic stem cells (Figures 2A–I). The findings demonstrated that the cultured BMSCs highly expressed the CD44 and CD29 markers, but did not express CD45, supporting that the cultured cells were BMSCs at passage four.

### Differentiation of BMSCs *In Vitro*

**Figure 3:**
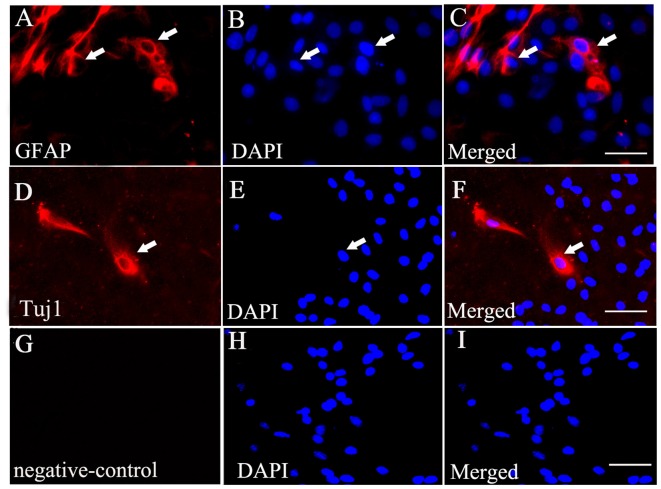
BMSCs could differentiate into neurons and astrocytes when cultured *in vitro*. **(A)** Immunofluorescent staining of glial fibrillary acidic portein (GFAP) that exhibits red fluorescence in the cultured BMSCs. **(D)** Immunofluorescent staining of Tuj1 showing red fluorescence in the cultured BMSCs. **(B,E,H)** Cell nucleus was stained by DAPI, with blue fluorescence. The merged images showed BMSC may have ability to differentiate into neurons **(F)** and astrocytes **(C)** under the 10% FBS condition. **(G,I)** Negative control (no primary antibody) showed no positive red staining, and there is only DAPI positive staining (blue). White arrows showed the positive cells. Bar = 50 μm. GFAP, glial fibrillary acidic protein; Tuj1, Neuronal Class III β-Tubulin.

Immunofluorescent staining showed that some of the BMSCs exhibited Tuj1-positive (Figures 3D–F) and GFAP-positive staining (Figures 3A–C) when exposed to culture medium containing 10% FBS. Additionally, no positive staining was identified in the negative control (no primary antibody; Figures 3G–I).

### Expression of NTFs *In Vitro*

**Figure 4:**
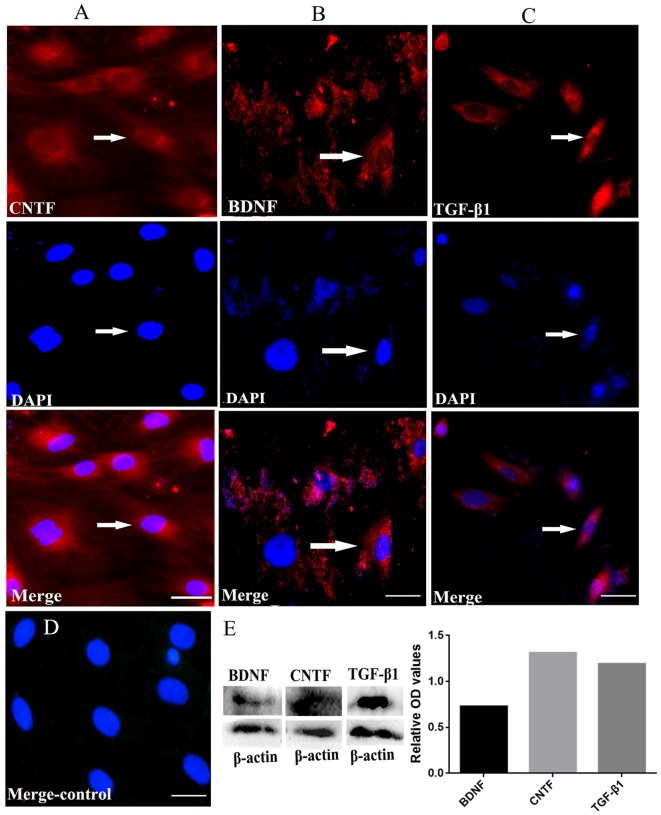
BMSCs could secrete NTFs *in vitro* revealed by immunofluorescent staining and western blots. (**A**, red, upper) Immunofluorescent staining of CNTF. (**B**, red, upper) Immunofluorescent staining of BDNF. (**C**, red, upper) Immunofluorescent staining of TGF-β1. (**A–C**, blue, middle) DAPI staining in each detection. The merged images for CNTF, TGF-β1 and BDNF were also showed in **(A–C)** respectively. **(D)** The merged negative control showed no positive fluorescence. Bar = 25 μm. White arrows represent the positive cells. **(E)** Western blots showed the true expression of NTFs including CNTF, TGF-β1 and BDNF secreted by the cultured BMSCs *in vitro*. BDNF, brain-derived neurotrophic factor; TGF-β1, transforming growth factor beta 1; CNTF, ciliary neurotrophic factor.

Immunofluorescent staining revealed that the cultured BMSCs at passage four were positive for CNTF, TGF-β1 and BDNF (Figures 4A–C). The negative control showed no positive staining (Figure 4D). Moreover, western blots confirmed the expression of these neurotrophins in the BMSCs at passage four, with the relative expression of BDNF, CNTF and TGF-β1 being 0.727882, 1.311528 and 1.192493, respectively (Figure 4E). These findings imply that the cultured BMSCs highly expressed CNTF, TGF-β1 and BDNF.

### *In Vivo* Effects of BMSC Transplantation on the Spinal Cord and Motor Function of Tree Shrews

**Figure 5:**
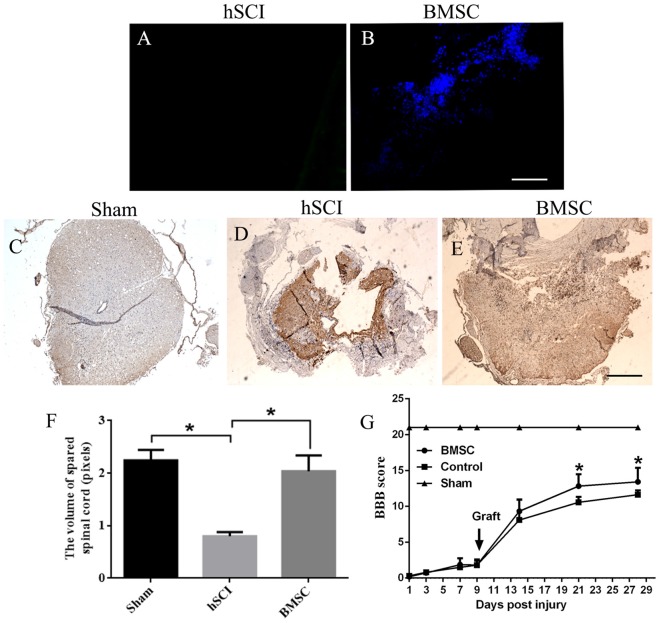
BMSC transplantation restored the volume of spared spinal cord and improved the Hind limb motor function of tree shrews after Hemi-sectioned spinal cord injury (hSCI). **(A)** Control group showed no positive staining. **(B)** BMSCs with Hoechst blue staining were widely diffused in the injured tissues at 28 days after injury. **(C)** Structure of the spinal cord in the sham tree shrews. **(D)** Structure of the spinal cord in the hSCI tree shrews. **(E)** Comparatively, BMSCs transplantation resulted in a significant increase on the volume of spared spinal cord. The pictures were taken at 1 cm below the lesion. Bar = 100 μm. **(F)** The bar chart for quantitative analysis of the volume of spared spinal cord among the sham, hSCI, and BMSC groups using Image-Pro plus 6.0 software. *p < 0.05 compared with the control. Data are presented as the mean ± SD (*n* = 5, one-way ANOVA). **(G)** Basso, Beattie, and Bresnehan (BBB) scores among the sham, control and BMSC groups at day 1–28 after injury. *p < 0.05 compared with the control. Data are presented as the mean ± SD (*n* = 10, one-way ANOVA). Arrowheads indicate the BMSC transplantation time. Control is the hSCI control group. BMSC group is hSCI + BMSCs group.

Hoechst 33342-stained BMSCs were detected in the tissues of the hSCI + BMSCs group, demonstrating the survival of the transplanted BMSCs (Figure 5B). In contrast, no Hoechst-positive cells were observed in the hSCI alone group (Figure 5A). At 28 days after injury, BMSC grafts promoted morphological improvement (Figures 5C–E). Quantitative analysis of the spinal cord volume revealed that the hSCI-induced reductions in spinal cord volume were not observed in the hSCI + BMSCs group (*p* < 0.05, Figure 5F); additionally, the volume in the hSCI + BMSCs group was not significantly different from that identified in sham animals (*p* > 0.05, Figure 5F). Evaluations of motor function showed that the BBB scores of the hSCI group were lower than those in the sham group. However, at days 21 and 28 after injury, the hSCI + BMSCs group had significantly higher BBB scores than did the hSCI alone group (*p* < 0.05; Figure 5G). These findings show that hSCI markedly damaged the spinal cord tissues and reduced the motor function of tree shrews, while BMSCs transplantation restored the reduced spinal cord volume after hSCI and improved motor function.

### *In Vivo* Effects of BMSC Transplantation on Scar Formation, IL-1β Expression and Cell Apoptosis

**Figure 6:**
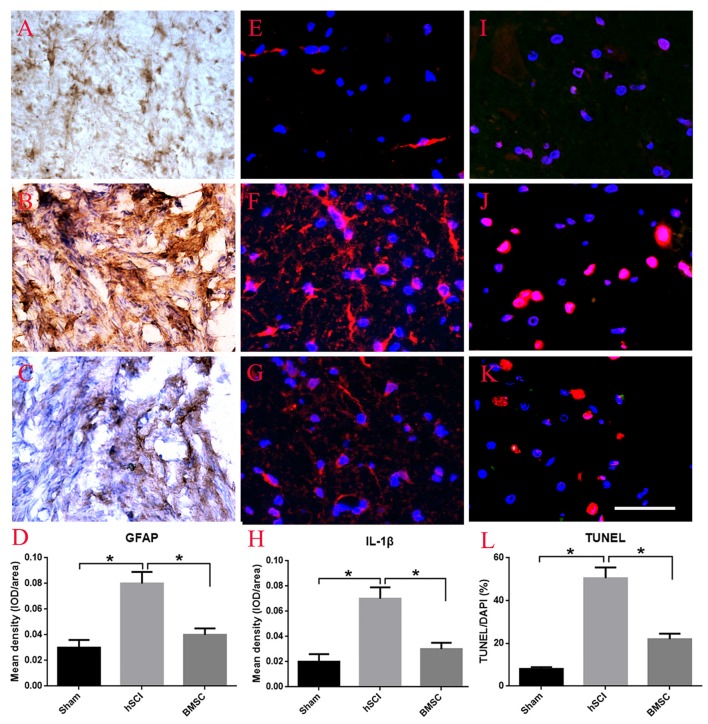
Effect of BMSC transplantation in reducing scar formation, anti-inflammation and apoptosis after hSCI in tree shrews. **(A)** Intact morphology of spinal cord in the sham group. **(B)** Scar formation labeled by GFAP staining in the hSCI group. **(C)** Scar formation labeled by GFAP staining in the BMSC transplantation group. **(D)** Quantitative analysis for the mean density of GFAP among the sham, hSCI, BMSC groups, which indirectly reflect scar formation. **(E–G)** Immuno-fluorescent staining of interleukin 1 beta (IL-1β) in the sham, hSCI, and BMSC groups. The mean density of IL-1β (IOD/area) is showed in **(H)**, which was analyzed using Image-Pro plus 6.0 software. **(I–K)** Cell apoptosis in the sham, hSCI, and BMSC groups. **(L)** Quantitative histogram representing the percent of terminal-deoxynucleoitidyl transferase mediated nick end labeling (TUNEL)/DAPI. *p < 0.05 compared with hSCI group. Data is presented as the means ± SD (*n* = 5, one-way ANOVA). Bar = 50 μm. BMSC group is hSCI + BMSCs group.

Using GFAP enzyme histochemical staining, we evaluated scar formation, which is indirectly reflected by the mean density of GFAP staining, in each group at 28 days after hSCI. In the sham group, some GFAP appearing yellow-brown positive staining was observed throughout the spinal cord (Figure 6A). After hSCI, the spinal cord was no longer intact and most of the GFAP-positive fibers were observed in the scar (Figure 6B). BMSC transplantation greatly reduced the density of GFAP staining, suggesting that transplanted BMSCs inhibited scar formation (*p* < 0.05, Figures 6C,D). Furthermore, we performed immunofluoscent staining for IL-1β to observe the effects of BMSCs on the inflammatory response. The results showed that compared to the hSCI alone group, the hSCI + BMSCs group displayed significantly less IL-1β staining in the spinal cord (*p* < 0.05, Figures 6E–H). As for cell apoptosis (the percent of TUNEL/DAPI), the hSCI + BMSCs group demonstrated less cell apoptosis than did the hSCI alone group (*p* < 0.05, Figures 6I–L). Collectively, these findings support that transplanting BMSCs into the spinal cord after injury reduces scar formation, inflammation and cell apoptosis.

### Effects of BMSC Transplantation on Motoneurons

**Figure 7:**
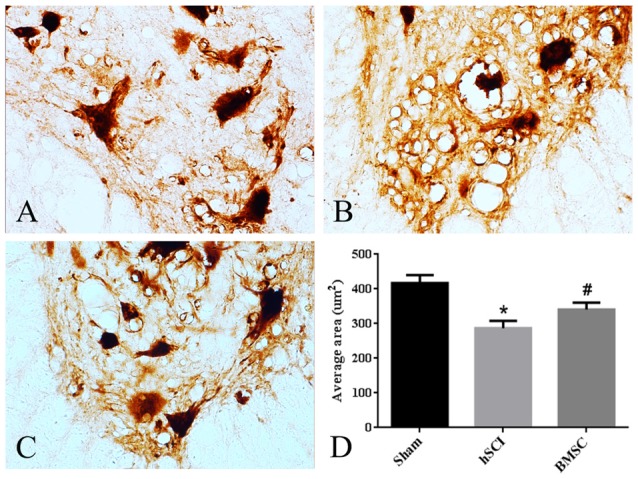
Morphological changes of motoneurons. **(A–C)** The morphology of motoneurons in ventral lamina IX. **(D)** Quantitative analysis for the area of the soma in motoneurons. Data are presented as the mean ± SD (*n* = 5, one-way ANOVA). *p < 0.05 compared with sham group. ^#^*p* < 0.05 compared with hSCI group. BMSC group is hSCI + BMSCs group.

To determine the neurotrophic effects of BMSC transplantation on motoneurons, we measured the area of the soma in motoneurons from lamina IX of the ventral horn. We found that the motoneurons appeared shrunken morphologically and that the average soma area was smaller in the hSCI alone group than it was in the sham group (*p* < 0.05). In contrast, the soma area of the hSCI + BMSC group was larger than was that of the hSCI group (*p* < 0.05) but was not significantly different from that of the sham group (*p* > 0.05; Figures 7A–D).

### Expressional Changes in *CNTF* and *STAT3* mRNA *In Vivo* after BMSC Transplantation

**Figure 8:**
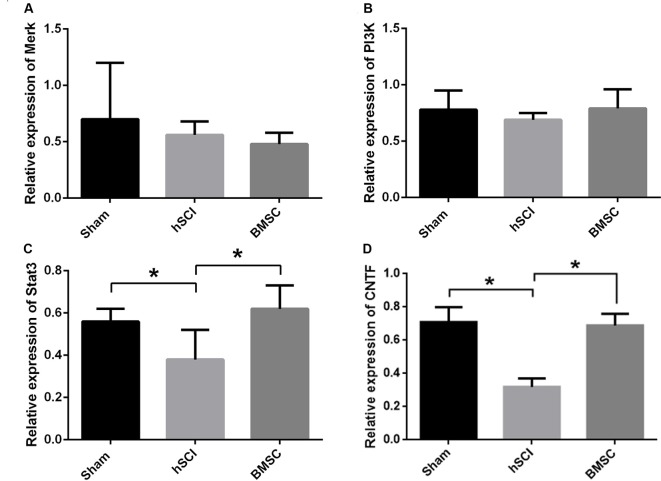
Verification of mitogen-activated protein kinase kinase (*Merk*), phosphatidylinositol 3-kinase (*PI3K*), signal transducer and activator of transcription 3 (*STAT3*) and *CNTF* mRNA levels after BMSC transplantation. Analysis of the *Merk* **(A)**, *PI3K* **(B)**, *STAT3* **(C)** and *CNTF* **(D)** mRNA levels among the sham, hSCI and BMSC group at 28 days after injury. *p < 0.05 compared with hSCI group. Data are presented as the mean ± SD (*n* = 5, one-way ANOVA). BMSC group is hSCI + BMSCs group.

At day 28 after injury, qRT-PCR showed that the mRNA expression of *CNTF* and *STAT3* in the hSCI alone group was significantly downregulated compared to that in the sham group (*p* < 0.05). The *CNTF* and *STAT3* mRNA levels in the hSCI + BMSC group were markedly upregulated (*p* < 0.05) compared to the hSCI alone group but were not significantly different from the levels in the sham group (*p* > 0.05). No significant differences in the *Merk* and *PI3K* mRNA levels were noted among the three groups. Therefore, BMSC transplantation significantly inhibited the hSCI-induced decline in *CNTF* and *STAT3* expression (Figures 8A–D).

## Discussion

**Figure 9:**
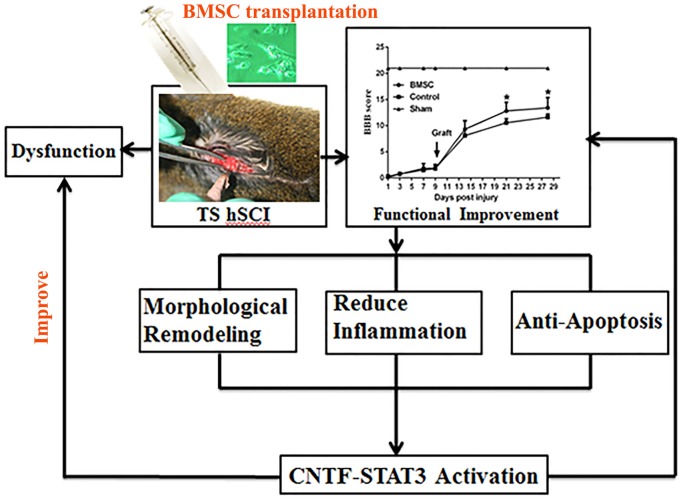
Flow chart of the whole experiment. Following hSCI, tree shrew exhibited a severe dysfunction in locomotor movement. However, transplantation of BMSCs with high capacity of proliferation resulted in a significant functional improvement. After that, morphological remodeling resulted from reduction of scar formation and inflammation as well as cell apoptosis may be the underlying mechanism of behavioral improvement. Furthermore, *CNTF*-regulated *STAT3* signaling may be the potential molecular events. In all, they eventually partly reverse the dysfunction and improve neural behavior.

The present study showed that BMSC transplantation improved the motor function of tree shrews after hSCI and that *CNTF*-regulated *STAT3* activation in the spinal cord may underlie this improvement (Figure 9).

### Characteristics of BMSCs *In Vitro* and *In Vivo*

The findings of the present study revealed that the purified BMSCs were able to grow, proliferate and partially differentiate into neurons or astrocytes in serum-induced conditions *in vitro*. In addition, Hoechst-labeled BMSCs were able to survive and migrate in tree shrews subjected to hSCI. These findings confirmed that BMSCs possessed the characteristics of stem cells and that they could be transplanted effectively. Previous studies have shown that BMSCs exhibit the capacity to self-renew and to differentiate into various cell lineages of mesenchymal origin, including chondrocytes, osteoblasts, adipocytes, cardiomyocytes, hepatocytes, endothelial and neuronal cells (Friedenstein et al., 1976). Additionally, BMSCs can suppress the immune response after transplantation and simultaneously maintain their multi-directional differentiation potential (Rossignol et al., 2009; Maggini et al., 2010). Together, these findings support that BMSCs may be a valuable tool for promoting nerve repair after SCI.

### BMSC Transplantation Promotes Functional Improvement in Tree Shrews Subjected to hSCI

Over the last few decades, GFP/DAPI/Hoechst-labeled BMSC transplantation has been shown to exert protective effects by promoting axonal regeneration, inhibiting cell apoptosis, and inducing the repair of nerve cells in rat models of radiation-induced multi-organ failure syndrome (Chapel et al., 2003), myocardial infarct (Shake et al., 2002), SCI (Zhou et al., 2013) and brain injury (Tanna and Sachan, 2014). Meanwhile, BMSCs were considered as vehicles, for gene delivery like GDNF or BDNF in the brain (Chen et al., 2003). However, as mentioned earlier, most of these studies were performed in rats. Since tree shrews share a more close relationship to primates than rodents (Fan et al., 2013, 2014), using this animal as a model may help reveal the potential therapeutic abilities of BMSC transplantation for humans. Therefore, in the present study, we used a tree shrew model to examine the effects of BMSC transplantation after hSCI. Our results showed that BMSC transplantation significantly protected the motorneurons and augmented the shrews’ BBB scores at day 21 and day 28 after injury, indicating that the grafted BMSCs improved the motor function of tree shrews with hSCI. Furthermore, BMSC transplantation significantly reduced glial scar formation, the inflammatory response and cell apoptosis in the tree shrew model of hSCI. Collectively, our findings demonstrated that BMSC transplantation had a positive effect on motor functional recovery after hSCI in tree shrews.

### Possible Molecular Events Involved in BMSC Transplantation

Here, we found that *CNTF* and *STAT3* expression was significantly upregulated in the hSCI + BMSCs group, while the expression of *Merk* and *PI3K* did not change. Previously, CNTF was shown to rescue various types of adult CNS neurons, including striatal, cholinergic forebrain, and dopaminergic midbrain neurons, as well as motoneurons, in several disease models (Hagg and Varon, 1993; Mitsumoto et al., 1994; Anderson et al., 1996), thus demonstrating that CNTF plays important protective roles in several diseases. As for SCI, one study found that *CNTF* and *CNTFR* mRNA expression increased dramatically at day 1 after hSCI in T2, then declined between days 1 and 5, and finally returned to normal by day 10, suggesting that CNTF may play a local role in the response to SCI (Oyesiku et al., 1997). In comparison, we found that *CNTF* mRNA expression in the area caudal to the SCI dramatically declined by 28 days after hSCI in T10; however, this reduction in *CNTF* mRNA expression did not occur in the hSCI + BMSCs group, as the level was similar to that observed for the sham group. Increasing evidence suggests that NTFs can alter the balance between neurite re-growth-inhibiting and -inducing molecules. As a result, these NTFs, particularly CNTF, can promote cell survival and axonal re-growth over neurodegeneration (Müller et al., 2009; Abbaszadeh et al., 2015). In addition, CNTF has been speculated to improve the efficacy of cell transplantation, highlighting the important role CNTF plays during BMSC transplantation in the rat SCI model (Abbaszadeh et al., 2015). Interestingly, STAT3, a downstream signaling molecule of CNTF, was also altered in our study, the results showed the expression level of *STAT3* was significantly upregulated after BMSC transplantation, indicating that endogenous synthesis of STAT3 was triggered. Moreover, in order to exclude the effects of immunosuppression on the *STAT3* mRNA levels, all tree shrews were treated with cyclosporin-A. Therefore, we speculate that BMSC transplantation improved motor function in tree shrews subjected to hSCI by increasing the *CNTF*-regulated *STAT3* signaling.

## Conclusion

This study demonstrated that BMSC transplantation into the injured spinal cord could improve motor function of tree shrews after hSCI, and that possible mechanisms are associated with *CNTF-*regulated *STAT3* signaling.

## Author Contributions

L-LX, FL and T-HW designed the experiments. L-LX, FL, B-TL, X-JD and JL performed the experiments. L-LX, PZ and R-PZ analyzed the data. L-LX, W-LZ and T-HW wrote the manuscript. All authors read and approved the final manuscript.

## Conflict of Interest Statement

The authors declare that the research was conducted in the absence of any commercial or financial relationships that could be construed as a potential conflict of interest.
